# Hypoxia responsive nano-drug delivery system based on angelica polysaccharide for liver cancer therapy

**DOI:** 10.1080/10717544.2021.2021324

**Published:** 2021-12-30

**Authors:** Xue Liu, Zhenfeng Wu, Chunjing Guo, Huimin Guo, Yanguo Su, Qiang Chen, Changgang Sun, Qingming Liu, Daquan Chen, Hongjie Mu

**Affiliations:** a Collaborative Innovation Center of Advanced Drug Delivery System and Biotech Drugs, School of Pharmacy, Yantai University, Yantai, PR China; b Key Laboratory of Modern Preparation of TCM, Ministry of Education, Jiangxi University of Traditional Chinese Medicine, Nanchang, PR China; c College of Marine Life Science, Ocean University of China, Qingdao, PR China; d Department of Oncology, Weifang Traditional Chinese Hospital, Weifang, PR China; e Shandong Academy of Chinese Medicine, Jinan, PR China

**Keywords:** Hypoxia responsive, angelica polysaccharide, micelle, liver cancer, ferroptosis

## Abstract

Based on the tumor hypoxic microenvironment and the new programmed cell death mode of combined ferroptosis, an angelica polysaccharide-based nanocarrier material was synthesized. The polymer contains hydrophilic angelica polysaccharide (ASP) that is linked by azobenzene (AZO) linker with ferrocene (Fc), and then the side chain was covalently modified with arachidonic acid (AA). It was postulated that the polymer micelles could work as an instinctive liver targeting drug delivery carrier, owing to the existence of ASP with liver targeting. Moreover, the aim was to engineer hypoxia-responsive polymer micelles which was modified by AA, for selective enhancement of ferroptosis in solid tumor, via diminishing glutathione (GSH) under hypoxia. Finally, we synthesized the amphiphilic polymer micelles AA/ASP-AZO-Fc (AAAF) by self-assembling. The structure of AAAF was confirmed by ^1^H-NMR and FT-IR. Then, we exemplified the hydrophobic medication curcumin into polymer micelles AAAF@Cur, which has smooth and regular spheres. In vitro release test affirmed that AAAF@Cur can achieve hypoxia response to drug release. In addition, a series of cell experiments confirmed that hypoxia could enhance cell uptake and effectively improve the proliferation inhibitory activity of HepG2 cells. In conclusion, AAAF, as an effective cell carrier, is expected to develop in sensitizing ferroptosis and anti-tumor.

## Introduction

Liver cancer, as a malignant cancer with high morbidity and high mortality (Llovet et al., 2018), is a great threat to the health of people all over the world. The main treatment methods are chemotherapy and surgical resection, but there are some problems, such as the lack of chemotherapeutic drug specificity, the damage to normal cells, and the high recurrence rate. Taking into account this phenomenon, it is urgent to seek an efficient treatment method.

Hypoxia, as an important microenvironment of solid tumors, creates a physical barrier. It is an imperative reason for angiogenesis, tumor metastasis, and enhancement of the drug resistance (Teicher, 1994; Harrison & Blackwell, 2004). This barrier will be a challenge for most anticancer drugs to reach the tumor site, which limits the therapeutic effect of drugs (Primeau et al., 2005). Following consideration of the limitation of the microenvironment of hypoxia on tumor treatment, two ways are proposed, which are ‘overcoming the hypoxia’ and ‘dodging the hypoxia’ (Hu et al., 2020). The former means increasing the oxygen content of tumor tissue; the latter refers to the use of water/gas molecules in tumor tissue to enhance the therapeutic effect of hypoxia tumors and weaken oxygen dependence. Apart from that two ways, the strategy of ‘using the hypoxia’ is accepted. A new nanocarrier including azobenzene (AZO) and nitroimidazole (Liu et al., 2015; Lee et al., 2017; Liu et al., 2017; Yang et al., 2019; Shen et al., 2021) can be constructed by using the oxygen-deficient response sensitive bond, so as to realize the stimuli-responsive release of the drug under the tumor microenvironment. So that it can greatly enhance the drug concentration at the tumor site and enhance the chemotherapy effect. In fact, azobenzene is a well-known example of the hypoxia-sensitive factor. The main chain of the carrier material can be easily broken relying on the reduction reaction in a low oxygen environment, and then triggers the release of the drug.

Natural polysaccharides, such as hyaluronic acid (HA) (Fang et al., 2019), astragalus polysaccharides (APS), angelica polysaccharides (ASP), and a series of plant polysaccharides, have attracted more and more attention due to their high biocompatibility during the construction of carrier materials. Angelica, as a medicinal and edible homologous plant, appears in a variety of traditional Chinese medicine prescriptions (Wang et al., 2020b; Guo et al., 2021). At the same time, as a traditional Chinese medicine, Angelica has its unique nature, taste, and meridian theory. It can target the liver, one of its essences lies in the existence of polysaccharide components. ASP is part of the principal active components in *Angelica sinensis*, which can enhance the body's immunity, anti-tumor activity, and has good liver targeting (Gao et al., 2017; Xiao et al., 2017). ASP showed a high affinity for the asialoglycoprotein receptor (ASGPR) (Guo et al., 2021), which is the most well-known target receptor in hepatocytes. In the light of this information, it is a good strategy to construct ASP-based nanoparticles through chemical modification to achieve liver targeting.

Different from apoptosis, necrosis, and autophagy, ferroptosis is an iron-dependent and a new type of programmed cell death (Stockwell, et al., 2017). There is increasing evidence that ferroptosis involves the application in a variety of diseases (Wang et al., 2010; Shen et al., 2018; Hassannia et al., 2019; Li et al., 2019), including acute kidney injury, tumor, and so on. The discovery of ferroptosis has become a new target for anti-tumor therapy (Yu et al., 2015). Expanded iron collection, creation of free extremists, supply of unsaturated fats, and lipid peroxidation are vital variables to instigate ferroptosis (Chen et al., 2021a,b). The vast majority of them are tumor development advancing components, which enormously expand the helplessness of malignancy to ferroptosis. Arachidonic acid (AA) can cause lipid peroxidation when it is presented to a high ROS environment in tumor, which causes glutathione (GSH) decrease, as a ‘deadly sign’ of ferroptosis (Gao et al., 2019). Notwithstanding the lipid peroxidation actuated by AA itself, iron additionally has solid support in it (Wang et al., 2018; Lei et al., 2020). Among them, ferrocene (Fc), as a stable organometallic complex, can produce ROS under physiological conditions, which are an ideal exogenous Fe^3+^ (Stockwell et al., 2017; Sang et al., 2019; Hou et al., 2020). On the one hand, the introduction of Fc can directly produce lipid peroxide by the Fenton reaction. On the other hand, ROS produced by the reaction can indirectly enhance the lipid peroxidation of AA and sensitize ferroptosis. The azobenzene mentioned above responds to the reductive degradation of the tumor hypoxia microenvironment, which requires the participation of NADPH. In the meantime, NADPH is also a benchmark for the normal antioxidant function of GSH (Guo et al., 2020). Therefore, selective consumption of NADPH at the tumor site can enhance the sensitivity of anti-tumor therapy based on ferroptosis.

Curcumin (Cur), as a traditional Chinese medicine extract, has a wide range of effects, including anti-inflammatory, anti-cancer, anti-oxidation, and so on (Hou et al., 2020). The poor water solubility of curcumin limits its clinical application (Wang et al., 2019). To improve the water solubility and bioavailability of curcumin, polymer micelles have been proposed to encapsulate curcumin in the hydrophobic core.

**Figure 1.**
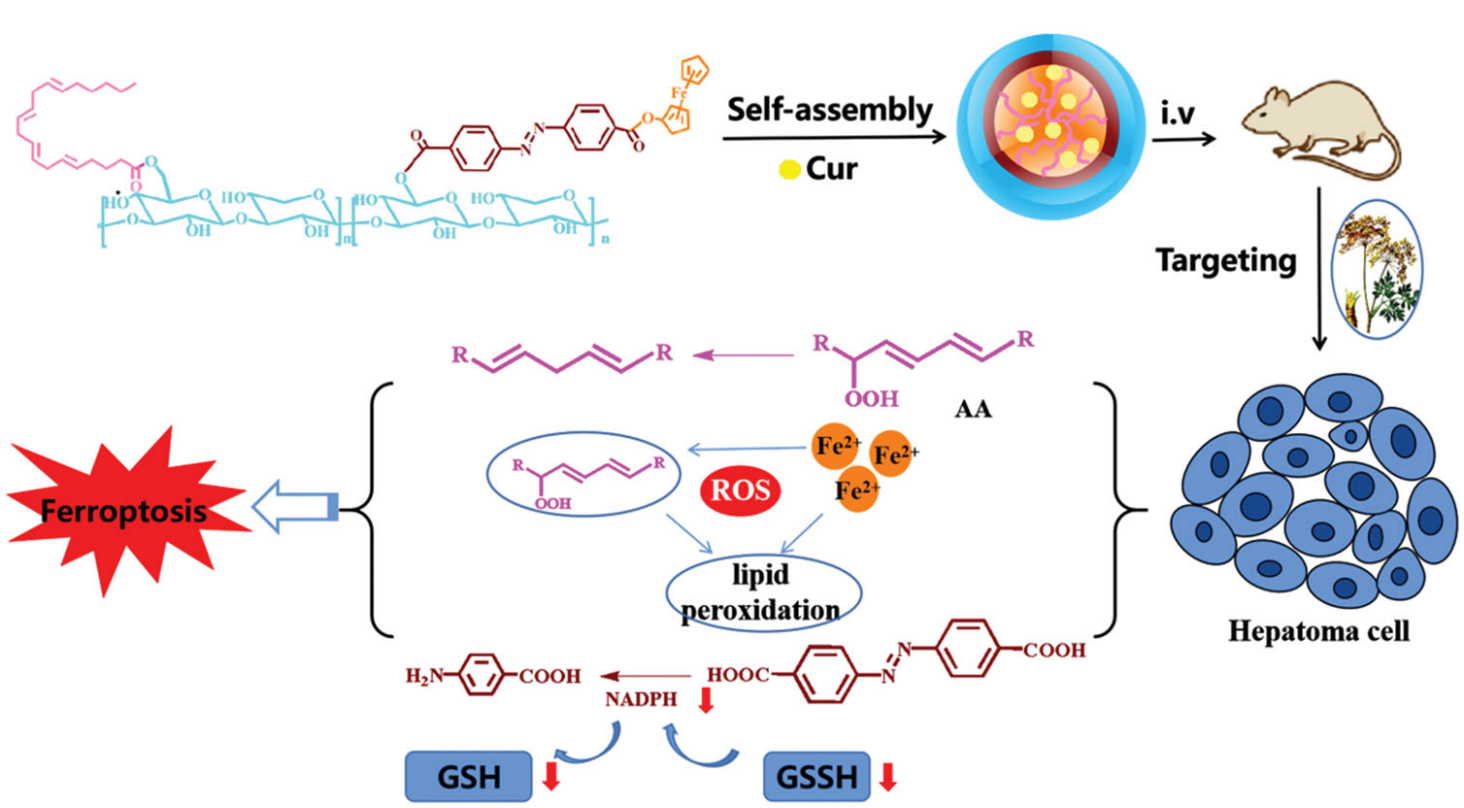
Design and schematic illustration of AAAF@Cur micelle.

Based on the inherent liver targeting of ASP, we took it as the hydrophilic end and connected the Fc with azobenzene linker to construct the amphiphilic carrier material AA/ASP-AZO-Fc (AAAF), to realize the construction of liver targeted nanoparticles with sensitized ferroptosis and antitumor effect (Figure 1). Co-delivery of drug with antitumor activity is to achieve synergistic improvement of antitumor effect. In addition, the introduction of AA and Fc can reduce the content of glutathione, enhance the sensitivity of ferroptosis, and improve the effect of anti-tumor therapy. Traditional Chinese medicine nano-targeted drug delivery system is proposed to realize the broad application prospect of anti-tumor.

## Materials and methods

### Materials

ASP was bought from Shanghai yuanye Bio-Technology Co., Ltd. Cur came from Zhanyun Chemical Co., Ltd (Shanghai, China). Fc was supplied from Beijing Solaibao Technology Co., Ltd. Sodium dithionite (Na_2_S_2_O_4_), azobenzene-4,4′-dicarboxylic acid (AZO), 1-(3-dimethylaminopropyl)-3-ethylcarbodiimide hydrochloride (EDC), 4-dimethylaminopyridine (DMAP), 1-hydroxybenzotriazole hydrate (HOBT), and AA were procured from Aladdin Chemistry Co., Ltd. CoCl_2_·6H_2_O was obtained from Sigma-Aldrich. Triethylamine (TEA) was purchased by Macklin Co., Ltd.

HepG2 cell line (human hepatocellular carcinoma) was obtained from Shandong Academy of Pharmaceutical Science. Human umbilical vein endothelial cells (HUVECs) were obtained from Hunan Fenghui Biotechnology Co., Ltd. Fetal bovine serum (FBS) was purchased from Zhejiang Tianhang Biotechnology Co., Ltd. DMEM medium for cell culture was provided from Thermo Fisher Scientific. MTT was obtained from Shanghai Yuanmu Biotechnology Co., Ltd.

### Synthesis of AA/ASP-AZO-Fc(AAAF)

**Figure 2.**
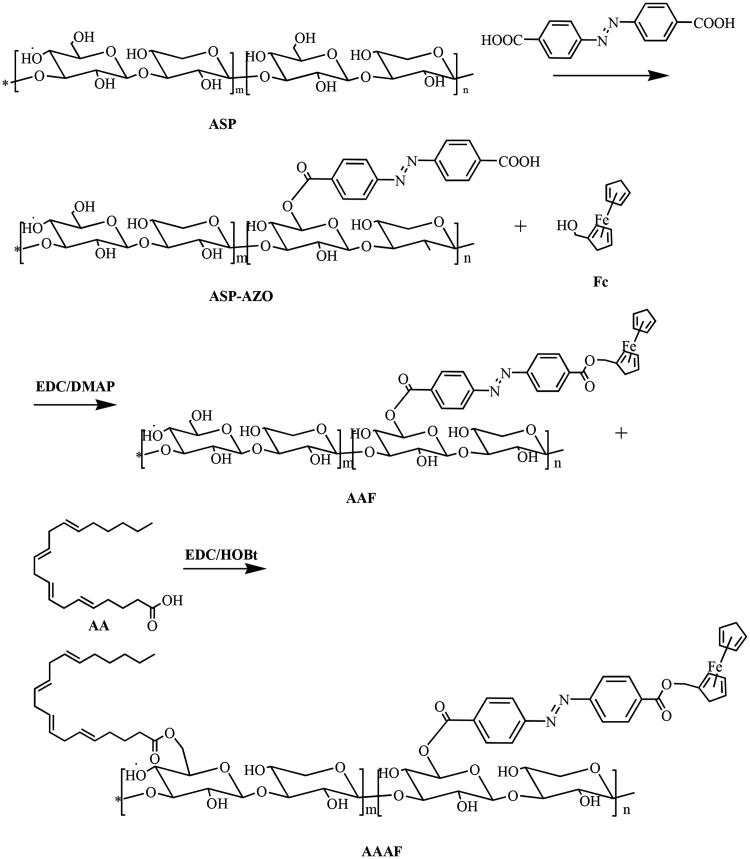
Synthetic route of ASP-AZO-Fc and AA-ASP-AZO-Fc.

The preparation route of AA/ASP-AZO-Fc (AAAF) is shown in Figure 2. The specific experimental steps were as follows.

#### Synthesis of ASP-AZO-Fc (AAF)

Step 1: AZO (54.048 mg, 0.18 mM), EDC (38.34 mg, 0.2 mM), and DMAP (21.99 mg, 0.18 mM) were mixed and dissolved in 5 mL DMSO, and then transferred to the reaction flask at 30 °C for 3 h. After the above reaction, 120 mg ASP was dissolved in 3 mL DMSO and added to the reaction flask. In the meantime, the mixtures were added 40 μL of TEA. We could get the product AZO-ASP after 24 h.

Step 2: Fc (41.60 mg, 0.18 mM) was dissolved in 2 mL DMSO, and then added to the above reaction flask. After that, adjusted the reaction temperature to 35 °C and continued the reaction for 5 h. The main chain compound ASP-AZO-Fc was obtained.

#### Synthesis of AA/ASP-AZO-Fc (AAAF)

EDC (57.51 mg, 0.3 mM), HOBt (36.52 mg, 0.3 mM), and 60 μL AA were mixed and dissolved in 2 mL of DMSO, and then transferred to the reaction flask at 30 °C for 2 h. After the activation, the reaction solution was transferred to the reaction bottle and continued for 5 h. Finally, the obtained mixed solution was transferred to the dialysis bag (2000 Da, MWCO), dialyzed in deionized water for another 24 h, and freeze-dried to obtain the final product AA/ASP-AZO-Fc (AAAF).

### Determination of critical micelle concentration

The critical micelle concentration (CMC) of the AAAF was determined by a well-established method using pyrene as a fluorescence probe. Acetone was used as a solvent, 100 μL pyrene solution was added into the centrifuge tube, and was evaporated at room temperature. Then, a series of carrier material aqueous solutions with different concentration gradients (.001, .002, .005, .01, .02, .05, 0.1, 0.2, 0.5, 0.8, 1.0 mg/mL) were prepared. One milliliter of the solution was added into a centrifuge tube, was placed on a water bath at 65 °C for 3 h, and kept overnight at room temperature. Two hundred microliters for every three parallel experiments (*n* = 3) were carried out in each group with 96-well plates. The excitation light luminescence was determined at 335 nm, the emission light 1 was at 373 nm, and the emission light 2 was at 384 nm. The absorbance was determined by a microplate reader (Thermo Fisher Scientific Co., Waltham, MA).

### The preparation of AA/ASP-AZO-Fc@Cur(AAAF@cur) micelles

Using Cur as the model drug, AAAF@Cur micelles were prepared by the self-assembly method. Ten milligrams of carrier material and 1 mg of free drug Cur were dissolved in 3 mL and 1 mL DMSO, respectively. The two solutions were mixed, and then transferred to 500 Da dialysis bags for 24 h. At the end of dialysis, the misture were centrifuged at 1000 rpm for 5 min, and filtered using 0.45 µm pore size filter. After that, we could obtain the micelle loaded with Cur (AA/ASP-AZO-Fc@Cur).

### Characterization of AA/ASP-AZO-Fc@Cur(AAAF@Cur) micelles

#### Particle size, zeta potential, and morphology of micelles under TEM

The particle size, potential, and PDI of AAAF@Cur micelles were determined by conventional instrument Delta Nano C (Beckman Coulter, IN). The morphology of the particles was characterized by transmission electron microscope (TEM, H-600; Hitachi, Tokyo, Japan). The degree of conjugation for AA and azobenzene was calculated on the basis of peak areas by ^1^H-NMR quantitative analysis of AAAF product.

#### Entrapment efficiency (EE%) and drug loading (DL%)

The drug loading and entrapment efficiency were established by UV-Vis (Shanghai Metash Instruments Co., Ltd, Shanghai, China). The micelle preparation was centrifuged and the supernatant was achieved. Methanol was added into 1 mL micelles for ultrasonic emulsification. After blank correction, the absorbance was determined at 425 nm. The following is a summary AAAF@Cur Methods for calculating drug loading and entrapment efficiency.

$$\mathrm{EE}(\%)\;=\frac{\left(\text{Mass of Cur in AAAF}@\text{Cur micelles}\right)}{\left(\text{Mass of all added Cur}\right)}\times 100\%$$

$$\mathrm{DL}(\%)=\frac{\left(\text{Mass of Cur in AAAF}@\text{Cur micelles}\right)}{\left(\text{Total Mass of the AAAF}@\text{Cur micelles}\right)}\times 100\%$$

#### Stability test

As amphiphilic materials, a key parameter for their applications as a nanocarrier is their stability. The stability test of AAAF@Cur micelles was examined in phosphate buffered saline (PBS) and fetal bovine serum (PBS) in the presence of 15% FBS solution using Delsa Nano C in different times (3 h, 6 h, 12 h, and 24 h).

### In vitro hypoxia response characteristics of micelles

To mimic the characteristics of micelles under hypoxia conditions in vitro, we chose Na_2_S_2_O_4_ as the reducing agent to simulate the hypoxic microenvironment. Hypoxia in tumor cells could promote the expression of nitroreductase, but the supplement of nitroreductase and NADPH alone in the buffer solution could not completely simulate the intracellular conditions, so the inorganic reductant was used for in vitro verification (Yan et al., 2019; Zhang et al., 2020).

#### Degradation of AAAF under a mimicked in vitro hypoxia environment

The placebo micelles (AAAF, 1 mg/mL, 4 mL) was mixed with different concentrations of Na_2_S_2_O_4_ (0 mM, 2 mM, 10 mM). After incubation at room temperature for 12 h, the UV-Vis absorption spectra were recorded.

#### In vitro drug release under mimicked hypoxia conditions

The release of AAAF@Cur in vitro was studied by the dialysis method. One milliliter of micelles was placed in a dialysis bag (2000 Da) and added to a 50 ml centrifuge tube. 40 ml of PBS (containing 0.7% Tween 80, pH = 7.4) were used as the release medium. Different masses of Na_2_S_2_O_4_ were added, adjusted to different concentrations (0, 10 mM, 20 mM, 30 mM), and vibrated at 37 °C. Samples were taken at different time points (1 h, 3 h, 5 h, 7 h, 9 h, 12 h, 24 h). One milliliter was taken each time, and the fresh medium of the same type and volume was added to control the constant volume. The concentration of Cur was determined, and the cumulative release in vitro was calculated. All groups included three parallel experiments.

$$Er\%=\frac{Ve\sum_{i=1}^{n-1}Ci+V0\mathrm{Cn}}{M\mathrm{drug}}\times 100\%$$

Each item in the formula is: *E*r% represents the cumulative release of the test drug; *V*e is the sample volume during the release process; *C*i is the release concentration of the drug during the sampling period; *V*_0_ is the volume of released medium in the centrifuge tube; *M*_drug_ is the mass of drug in cur-loaded AAAF micelles.

### Cell culture

HepG2 cells and HUVECs were used to evaluate the preparation in vitro. HepG2 cells and HUVECs were cultured in DMEM, which contains 10% FBS and were incubated at 37 °C in an incubator with 5% CO_2_ atmosphere.

### Intracellular GSH measurement

HepG2 cells (1 × 10^6^ cells/well) were seeded in six-well plates, and cultured overnight. And then, they were cultured under hypoxia or normoxia. After 24 h, HepG2 cells were co-incubated with placebo AAAF (100 μg/mL) for 2, 4, 6, 12, 24 h. The cells were digested by pancreatin, PBS cleaning, and then collected in a 15 mL centrifuge tube, centrifuged at 1000 rpm for 5 min. Moreover, cells without cell treatment were used as the control. The subsequent experimental process was based on GSH and GSSG detection Kits instruction (beyotime, Nantong, Jiangsu, China).

### In vitro cytotoxicity test

The cytotoxicity values of free Cur, AAAF, and AAAF@Cur micelles were evaluated against HepG2 by the MTT method. In this assay, HepG2 cells were seeded in a 96-well plate at a density of 6 × 10^3^ cells/well. Then, HepG2 cells were co-incubated with different concentrations of free Cur and AAAF@Cur. The wells were incubated at 37 °C for 24 h, and the wells without drug administration were used as blank control. Twenty microlitres of MTT were added (5 mg/ml) to each well. After 4 h, the original culture medium was taken the place of DMSO (150 μL), shaken at 37 °C for 10 min. The microplate reader (Thermo Fisher Scientific Co., Waltham, MA) was used to determine the absorbance at 490 nm. Moreover, the cytotoxicity of free drugs and micelles was studied in a hypoxia microenvironment. HepG2 cells (10^4^ cells/well) were seeded in 96-well plates for 24 h. After that, it was transferred to the incubator for hypoxia treatment for 12 h, and the following steps were the same as above. In addition, we also tested the cytotoxicity of AAAF to HUVECs.

### In vitro cellular uptake and localization

#### Cellular uptake of time-dependent and hypoxic response micelles

HepG2 cells (8 × 10^4^ cells/well) were seeded in 12-well plate cultured overnight. The cells were treated with or without 200 μM CoCl_2_ for 24 h (Piret et al., 2002; Duan, et al, 2014; Zhou et al., 2018). Then, the medium was replaced with free Cur and AAAF@Cur micelles (Cur concentration: 20 μg/mL). After the predetermined time point (1 h, 2 h, 4 h), cells were washed with PBS three times, fixed with 4% paraformaldehyde for 20 min, and then washed with PBS twice. The results were observed by inverted fluorescence microscope.

#### Cell localization experiment

HepG2 cells were treated with AAAF@Cur (20 μg/mL) for 4 h. After the above treatment, 1 mL 4% paraformaldehyde was added for 20 min, and then washed with PBS three times. Cell nuclei were stained with DAPI for 15 min and observed by inverted fluorescence microscope.

#### Validation of specific liver targeting

In order to verify the specific liver targeting of ASP, ASGPR-poor HUVECs were used as control cells to observe the cell uptake after micelle treatment for 2 h and 4 h.

### Statistical analysis

All investigations were performed no less than multiple times and showed as mean ± standard deviation (SD). T-test and ANOVA were utilized to examine the information. Differences were considered statistically significant when *p*-values were less than .05. The intensity of all fluorescence expressions in the experiments was further calculated by Image J software.

## Results

### Characterization of AAAF

#### ^1^H-NMR

**Figure 3.**
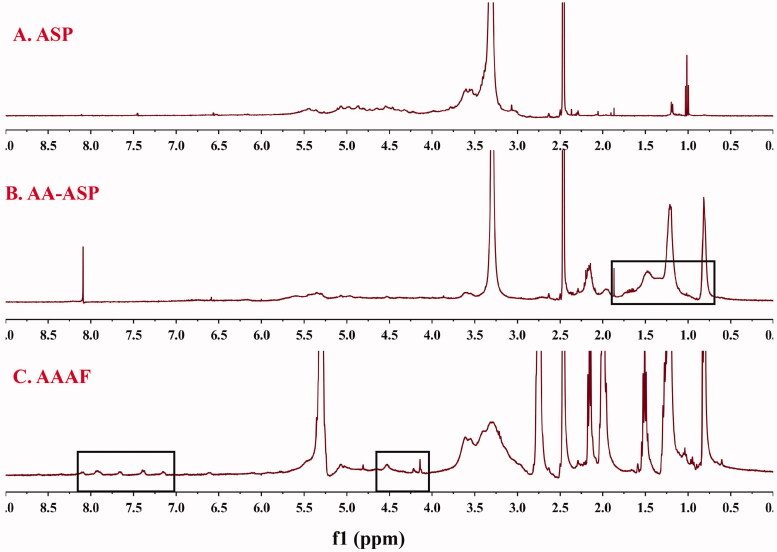
The ^1^H-NMR spectra of ASP (A), AA-ASP (B), and AAAF (C).

As shown in Figure 3(B), the ^1^H-NMR spectra for AA-ASP showed the characteristic peaks of ASP between 2.5 and 5.7 ppm. Compared with Figure 3(A), the new peak of 0.5–1.5 ppm was the characteristic peak of -CH_3_ in AA. As shown in Figure 3(C), the characteristic peaks of hydrogen on the benzene ring of AZO were at 7.381 and 7.911 ppm. The typical signals at 4–5 ppm in Figure 3(C) were assigned to Fc, which confirmed that AAAF was obtained successfully.

#### FT-IR

**Figure 4.**
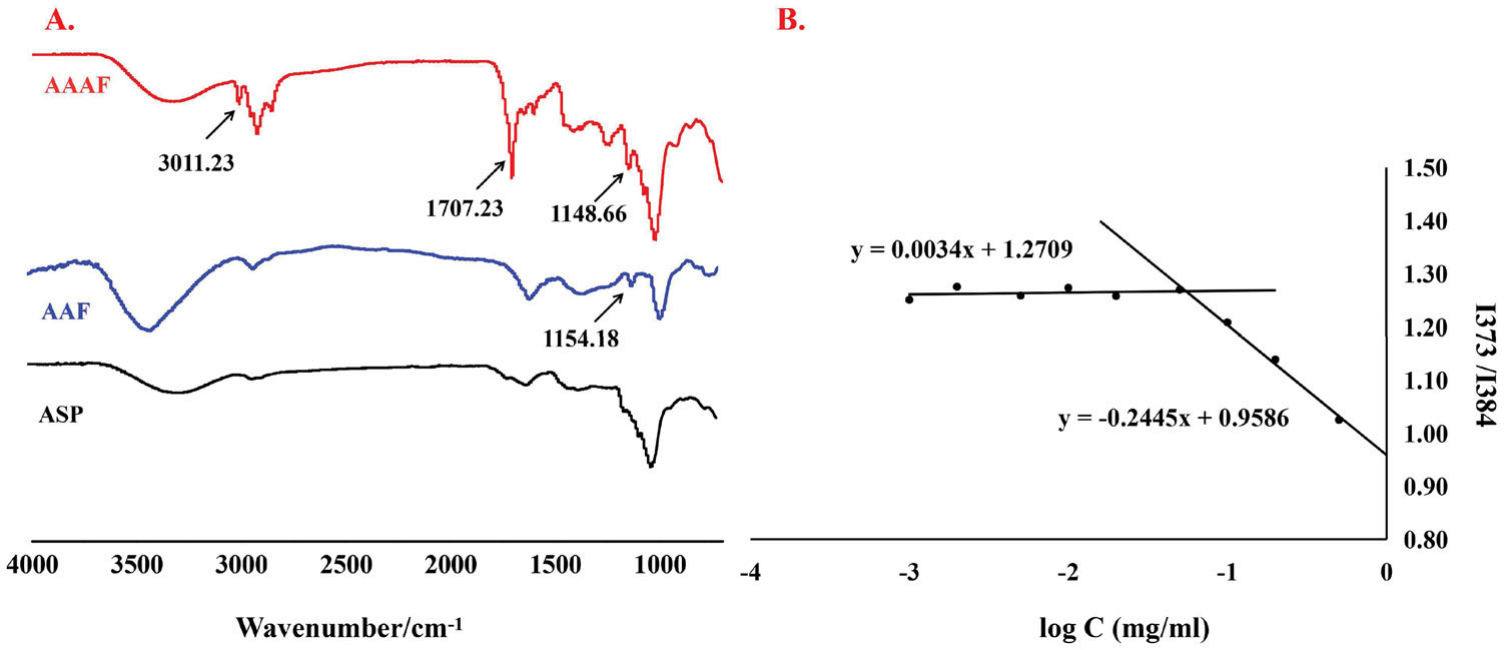
(A) The FT-IR spectrum of ASP, AAF and AAAF. (B) The CMC curve of AAAF micelles.

The infrared spectra of ASP, AAF and AAAF are shown in Figure 4(A). The broad band at 3000–3700 cm^−1^ was caused by the Tensile vibration of hydroxyl (–OH). Compared with ASP, in the AAAF spectrum, the newly stretching vibration band at 1707.23 cm^−1^ was attributed to C=O; the peaks at 2925.59 cm^−1^ belonged to the olefin double bond (C=C) indicated that AA was connected successfully. The characteristic peak at 3011.23 cm^−1^ and 1148.66 cm^−1^ were from Fc, indicating the introduction of Fc. In conclusion, the carrier material AAAF was successfully synthesized. Moreover, in the infrared spectra of AAF, the peaks at 1700 cm^−1^ and 1154.18 cm^−1^ which is equivalent to was attributed to the 1707.23 cm^−1^ and 1148.66 cm^−1^ in AAAF, indicating the successfully synthesis of AAF.

### Determination of CMC

The CMC of AAAF was determined by the pyrene fluorescence method. The results are shown in Figure 4(B). The CMC of AAAF was calculated to be 0.055 mg/mL, that is, the micelle concentration formed by the association of the carrier material in the solvent was at least 0.055 mg/mL.

### Characterization of AAAF@Cur micelles

#### Particle size, potential, electron microscopy, drug loading (DL%), and entrapment efficiency (EE%)

**Figure 5.**
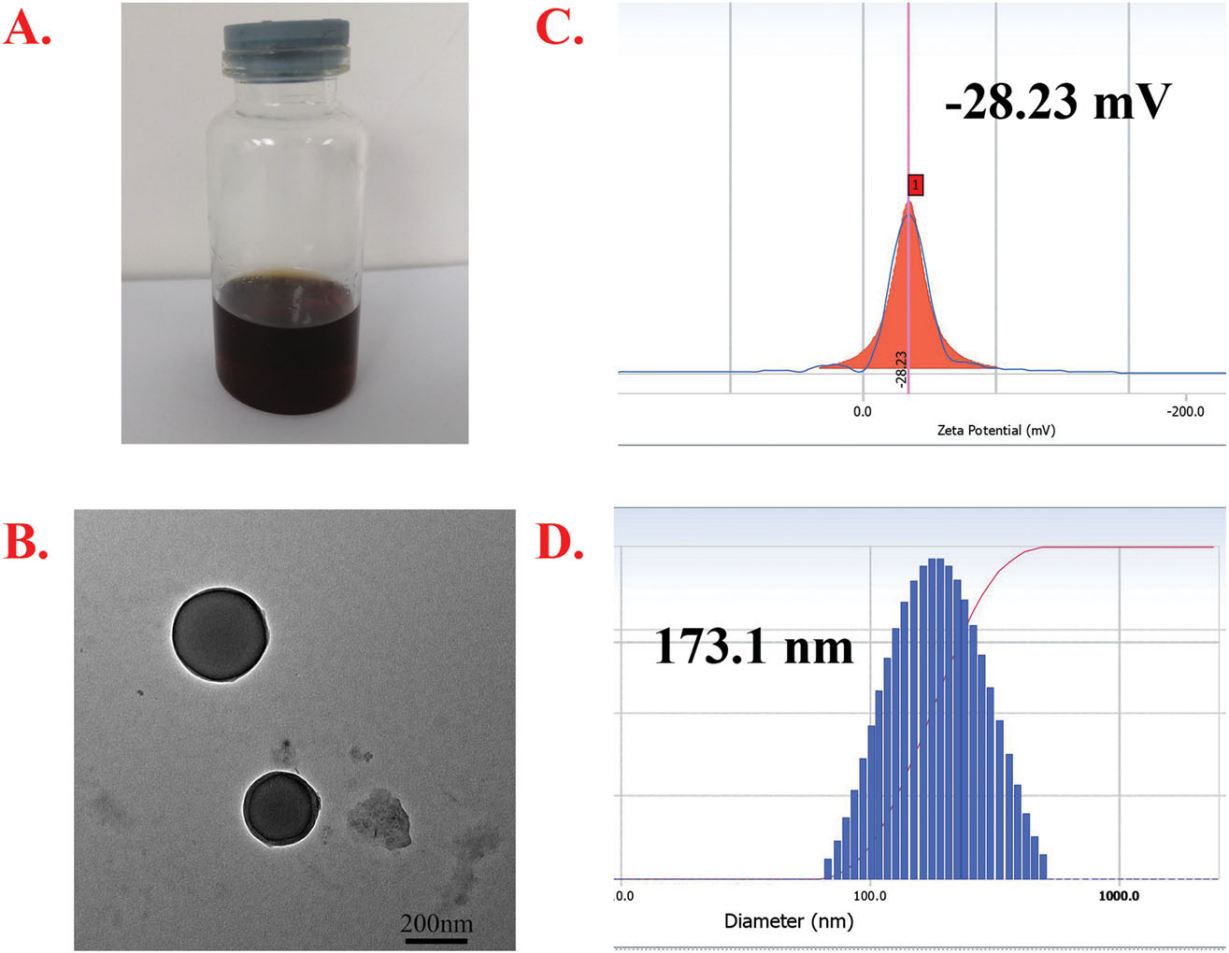
Appearance. (A) The TEM picture (Scale bar: 200.0 nm). (B) Zeta potential. (C) and particle size diagram. (D) Characterization of AAAF@Cur micelles.

The particle size, PDI, zeta potential, and TEM morphology are presented in Figure 5. The EE% and DL% of Cur were 45.9% and 4.97%, respectively. The dialysis method was used for preparation, and the appearance of the obtained micelles is shown in Figure 5(A). The structure of the micelle preparation was observed by TEM (Figure 5(B)). Under the TEM, the micelles were smooth, regular spherical, and well dispersed. The particle size and potential of the micelles were measured by Delsa Nano C. The results showed that the particle size was 194.5 nm, the potential was −28.23 mV, and the PDI was 0.102, suggesting that the prepared nanoparticles were much more uniform. The degree of conjugation for AA and azobenzene was 19.49% and 10.53%, respectively.

#### Stability test

As shown in Table 1, there was no significant change in the particle size of AAAF@Cur micelles in PBS and PBS containing 15% FBS. According to our review and practice, it was proved that the drug-loaded AAAF@Cur exhibited good stability in different dispersing media.

**Table 1.** The stability of AAAF@Cur Micelle.

Time (h)	PBS (nm)	PBS + 15% FBS (nm)
3	194.7 ± 0.5	202.0 ± 1.3
6	202.6 ± 1.1	206.6 ± 0.9
12	215.4 ± 0.6	224.5 ± 2.1
24	225.0 ± 1.6	245.3 ± 4.2

PBS: FBS: Fetal bovine serum.

### In vitro hypoxia response characteristics of micelles

#### Degradation of AAAF under a mimicked hypoxia environment in vitro

**Figure 6.**
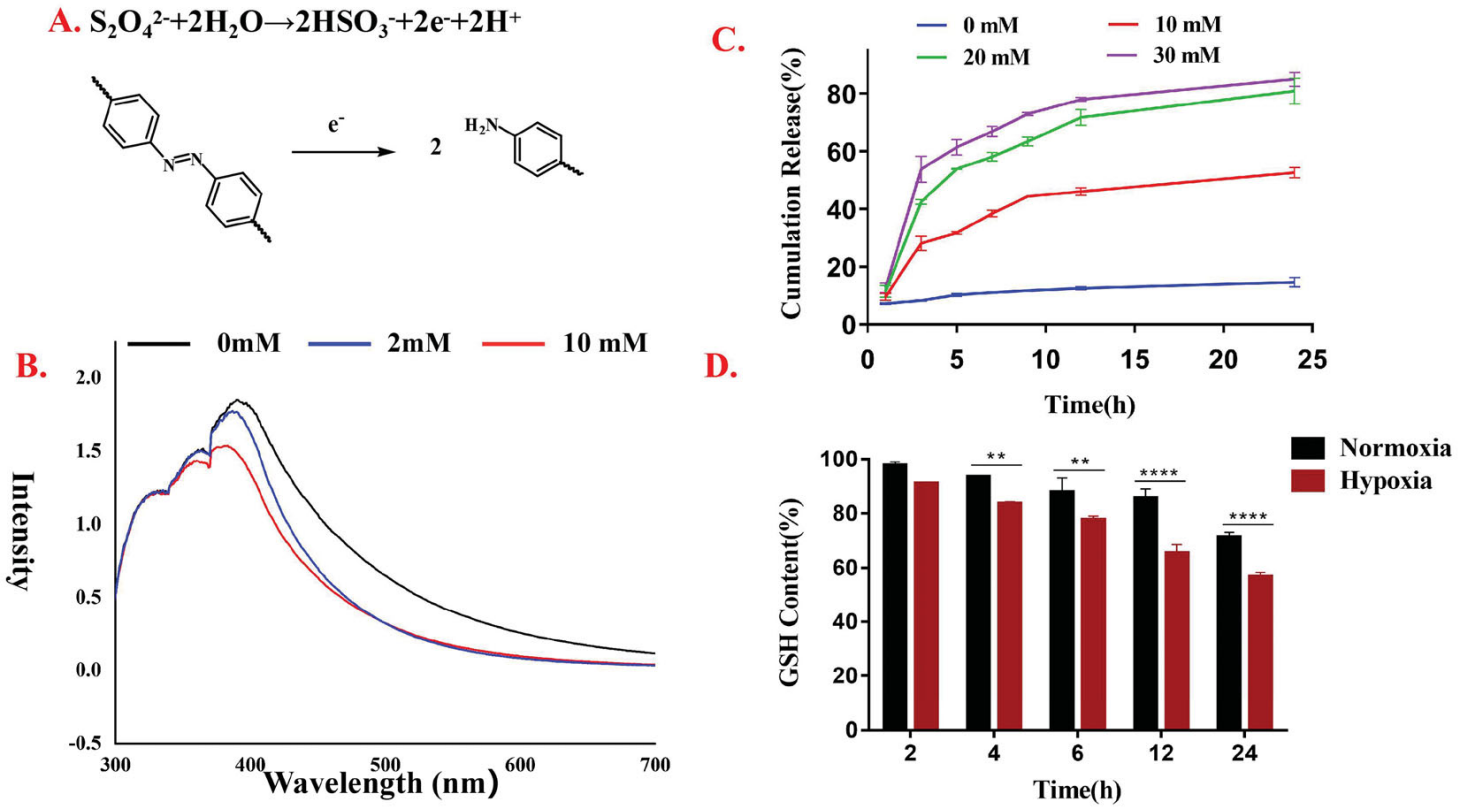
(A) The conversion of Azobenzene bond of AAAF micelles during Na_2_S_2_O_4_ treatment; (B) The UV-Vis spectroscopy of AAAF micelles treated by various concentrations of Na_2_S_2_O_4_ (0, 2, 5, 20 mM); (C) Cumulative release of Cur from AAAF@Cur under mimicked hypoxic conditions; (D) GSH level after treatment with placebo micelles AAAF. Data are shown as the mean ± SD (*n* = 3), *p < .05, **p < .01, and ***p < .001.

Na_2_S_2_O_4_ was incubated with blank micelles to simulate the hypoxic microenvironment in vitro. After incubation at room temperature for 12 h, the UV-Vis spectra were recorded (Figure 6(B)). The results showed that the UV absorption based on the AZO bond decreased significantly, and the decrease degree was proportional to the concentration of Na_2_S_2_O_4_. That was to say, the degradation degree of micelles was proportional to the concentration of Na_2_S_2_O_4_.

#### In vitro drug release under mimicked hypoxia conditions

The results of in vitro hypoxia-sensitive Cur release kinetics are presented in Figure 6(C). The drug was released at 37 °C in PBS containing Tween 80 (0.7%, w/w) and different concentrations of Na_2_S_2_O_4_ (SDT, a mimic reductant for azoreductase), which was used to mimic the hypoxia condition. At predetermined time points, samples were taken for the determination of Cur concentration. The cumulative drug released percent was plotted against time (*n* = 3). In the normoxia environment, the drug release was relatively slow, and the cumulative release was only up to 14.6%. However, in the presence of Na_2_S_2_O_4_, Cur was released rapidly with the increase of Na_2_S_2_O_4_ concentration, suggesting the micelles could release in response to hypoxia.

### Intracellular GSH measurement

GSH depletion was a marker of ferroptosis. As shown in Figure 6(D), the content of GSH decreased gradually for several reasons. Fc also contributed to the depletion of GSH. Fc promoted ROS level increase, and the ROS production consumed GSH, leading to GSH depletion. In addition, this was attributed to the lipid peroxidation of AA under the influence of high ROS, which leads to the reduction of intracellular GSH, thus enhancing the effect of ferroptosis on the removal of tumor cells. Besides, compared with the normoxia-treated group, the GSH content of cells treated with placebo micelle AAAF under hypoxia condition decreased significantly. It was presumed that the degradation of AZO could also be used as an inducement, resulting in the reduction of NADPH and the depletion of GSH.

### In vitro cytotoxicity test

**Figure 7.**
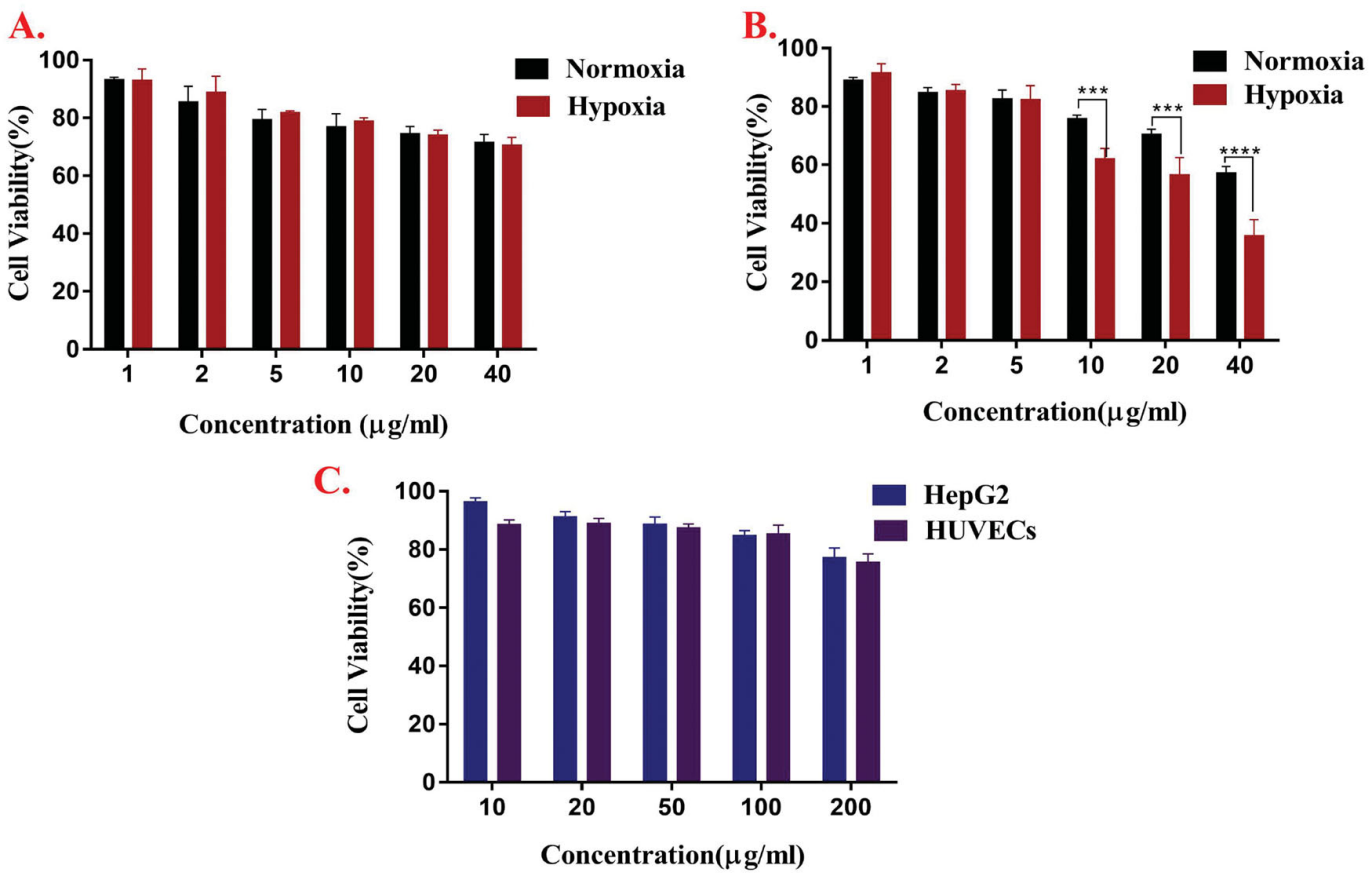
The cell viabilities of free Cur (A) and AAAF@Cur micelles (B) at 24 h in HepG2 cells under hypoxia and normoxia. (C) Cytotoxicity analysis of AAAF after incubation for 24 h on HepG2 and HUVECs. Data are shown as the mean ± SD (*n* = 3), *p < .05, **p < .01, and ***p < .001.

MTT method was used to evaluate the cytotoxicity of free Cur, AAAF, and AAAF@Cur micelles. As shown in Figure 7(A,B), it was the toxicity evaluation of Cur and AAAF@Cur on HepG2 cells for 24 h under normoxia and hypoxia. The results showed that the cytotoxicity of Cur and AAAF@Cur was dose dependent. Compared with normoxia condition, micelles treated with hypoxia condition have more obvious cytotoxicity. It was estimated that micelles had a more effective inhibitory effect on tumor cells, which was consistent with the theoretical results. Based on the responsive release of micelles in the tumor hypoxic microenvironment, it accelerated the release of drugs, enhanced the local drug concentration of hypoxic tumor cells, significantly improved the drug toxicity, and enhanced the therapeutic effect on tumors. It was evident that the cell viabilities of HepG2 cells and HUVECs (Figure 7(C)) were still above 85% after AAAF treated with 24 h, which indicated excellent safety and biocompatibility.

### In vitro cellular uptake and localization

**Figure 8.**
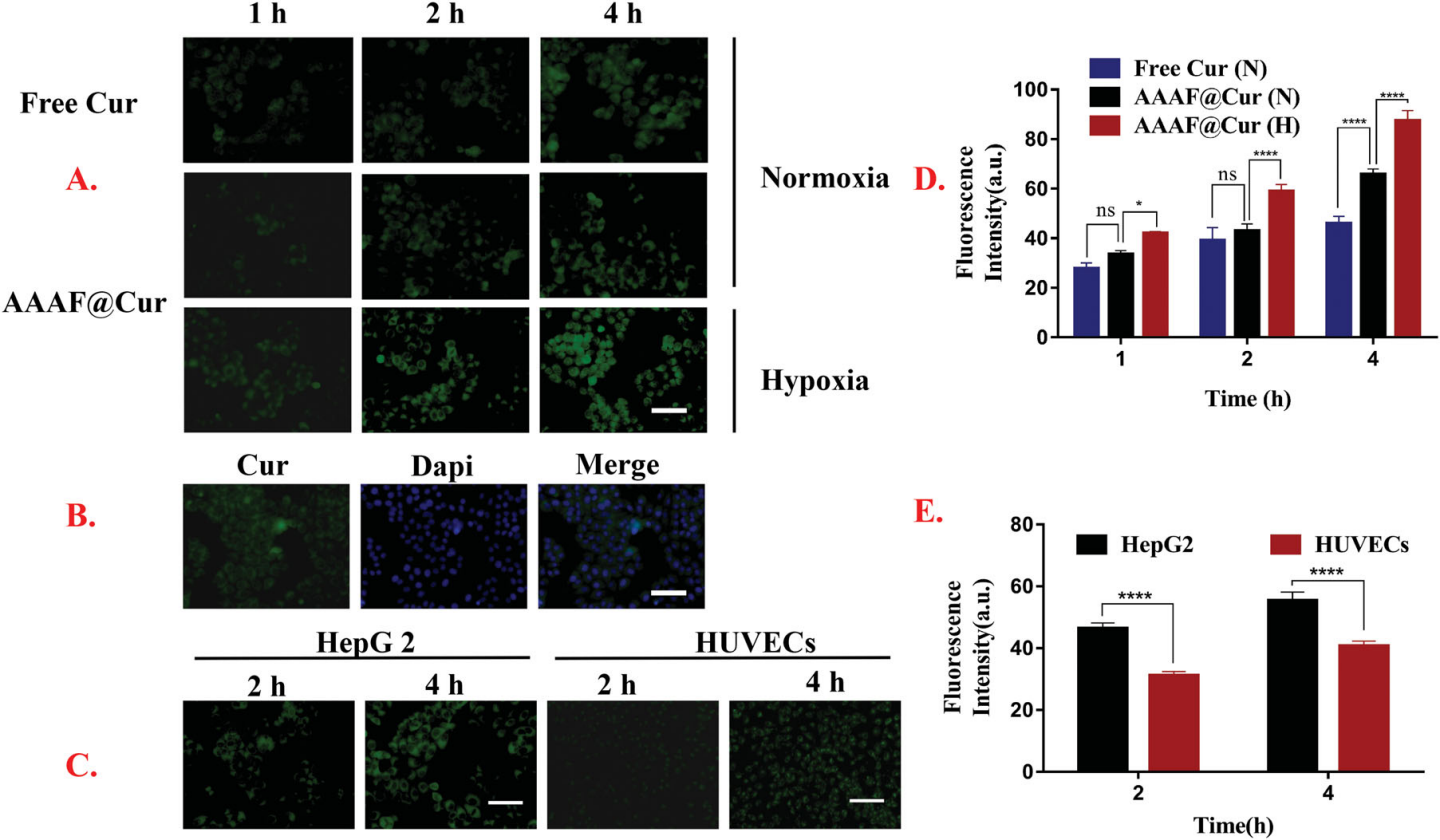
(A, D) Kinetic uptake and quantitative analysis of free Cur, fluorescent micelles by HepG2 cells under normoxia or hypoxia. (B) The cell localization of AAAF@Cur. (C, E). Kinetic uptake and quantitative analysis of HepG2 cells and HUVECs cells incubated with AAAF@Cur formulations for 2 and 4 h. Scale bar: 100 μm. Data are shown as the mean ± SD (*n* = 3), *p < .05, **p < .01, and ***p < .001. n.s.: no significant difference.

Effective cellular uptake was the key to evaluate the effect of drug preparations. In this experiment, based on its green fluorescence, free Cur was used as a fluorescent marker in addition to the model drug. The results (Figure 8(A,D)) showed that the cellular uptake of micelles increased with time. This result was consistent with the liver targeting of ASP. Moreover, the uptake of AAAF@Cur was markedly elevated under hypoxia treatment than that of normoxia condition at the same time, which indicated that the cellular internalization of micelles was outstanding under normoxia condition. This phenomenon could be explained by the reduction of azobenzene caused by hypoxia and the subsequent break of the main chain of polymer micelles, which resulted in the release of drugs and thus accelerated the uptake of drugs. Figure 8(B) shows that after DAPI labeling, the nucleus showed blue fluorescence, and AAAF was mainly absorbed by the cytoplasm, showing green fluorescence near the nucleus. In addition, in order to further verify the liver targeting of ASP micelles, we selected ASGPR-poor HUVECs as the control. Compared with HepG2 cells, weak fluorescence intensity was observed after micelle treated with HUVECs cells (Figure 8(C,E)). The results showed that ASP NPs could specifically target HepG2 cells, via the ASGPR-mediated endocytosis pathway.

## Discussion

Polysaccharides of Chinese traditional medicine have received considerable attention as an abundant resource to be utilized in drug development and disease treatment (Cao et al., 2018; Wang et al., 2020a). ASP, as one of the main polysaccharides extracted from the Angelica root, has anti-tumor and anti-inflammatory activities. At the same time, ASP has a high affinity for ASGPR and MR of hepatoma carcinoma cells (Zhang et al., 2018; Du et al., 2020), so it is expected to become a natural product for the preparation of liver-targeted carrier materials (Du et al., 2020). In this paper, an ASP-based nanocarrier was prepared by a self-assembling method in order to achieve liver targeting. With the introduction of ASP, the amphiphilic polymer micelles can specifically target the liver and reduce the damage to normal cells.

After the drug is targeted to the liver cancer site, based on its hypoxic microenvironment, it induces the rupture of AZO, a sensitive bond in response to stimulation, so that it enhances the release of drugs at the tumor site and improves the drug concentration at the tumor site. Hypoxia is one of the most significant characteristics of solid tumors. Its degree of hypoxia is closely related to the local concentration of reducing substances such as AZO. As a hypoxia-responsive group, AZO will undergo reductive cleavage under hypoxia conditions (Ma et al., 2019), break the polymer main chain, and then accelerate the release of drugs. The inhibitory effect of each preparation on tumor shows the significant therapeutic effect of hypoxia-responsive targeted drug delivery system. At the same time, the reduction of AZO group will cause the depletion of NADPH in cells, thus reducing the concentration of GSH in cells, indirectly promoting ferroptosis, and promoting the inhibitory effect of the preparation on tumor under hypoxic conditions. This result is similar to the decrease of intracellular GSH content under hypoxia.

In addition, as a new type of programmed cell death, ferroptosis plays an important role in the treatment of liver cancer, kidney cancer, and other types of cancer (Wang et al., 2020c). The existence of AA and AZO synergistically reduces the content of GSH and selectively sensitizes ferroptosis, which is expected to achieve efficient treatment of tumors.

## Conclusion

The introduction of active components of traditional Chinese medicine is an important development direction of nanomedicine. In this paper, a hypoxia-responsive and ASP-based liver targeting carrier material was successfully synthesized. The structure of the product was confirmed by ^1^H-NMR. Cur-loaded micelles prepared by dialysis method had good morphology, ideal particle size, and potential. In addition, in vitro release experiments showed that the release of the drug was a continuous process and increased with the concentration of Na_2_S_2_O_4_. The results of the GSH content index showed that micelle preparation treatment could significantly inhibit GSH content, and promote the occurrence of ferroptosis. In the aspect of cell experiments, a series of characterizations were carried out on the toxicity and targeting uptake ability of the preparation to HepG2 cells and HUVECs.

The results showed that the carrier material had excellent biocompatibility and had a liver-specific targeting effect compared with ASGPR-poor HUVECs. In addition to targeting liver cells and achieving the responsive release of the tumor microenvironment, the introduction of AA and Fc is expected to make ferroptosis synergistic anti-tumor therapy possible and has broad application prospects.

## Data Availability

The authors confirm that the data supporting the findings of this study are available within the article [and/or] its supplementary materials.
